# Differences in the Effects of Broad-Band UVA and Narrow-Band UVB on Epidermal Keratinocytes

**DOI:** 10.3390/ijerph182312480

**Published:** 2021-11-26

**Authors:** Robert Bajgar, Anna Moukova, Nela Chalupnikova, Hana Kolarova

**Affiliations:** 1 Department of Medical Biophysics, Faculty of Medicine and Dentistry, Palacky University in Olomouc, Hnevotinska 3, 775 15 Olomouc, Czech Republic; anna.moukova01@upol.cz (A.M.); nela.chalupnikova01@upol.cz (N.C.); hana.kolarova@upol.cz (H.K.); 2 Institute of Molecular and Translational Medicine, Faculty of Medicine and Dentistry, Palacky University in Olomouc, Hnevotinska 3, 775 15 Olomouc, Czech Republic

**Keywords:** UV radiation, photoageing, reactive oxygen species, DNA damage

## Abstract

Background: The sun is a natural source of UV radiation. It can be divided into three bands, UVA (315–400 nm), UVB (280–315 nm) and UVC (100–280 nm), where the radiation up to 290 nm is very effectively eliminated by the stratospheric ozone. Although UV radiation can have a beneficial effect on our organism and can be used in the treatment of several skin diseases, it must primarily be considered harmful. Methods: In the presented work, we focused on the study of the longer-wavelength UV components (UVA and UVB) on the human epidermal keratinocyte line HaCaT. As UVA and UVB radiation sources, we used commercially available UVA and UVB tubes from Philips (Philips, Amsterdam, The Netherlands), which are commonly employed in photochemotherapy. We compared their effects on cell viability and proliferation, changes in ROS production, mitochondrial function and the degree of DNA damage. Results: Our results revealed that UVB irradiation, even with significantly lower irradiance, caused greater ROS production, depolarization of mitochondrial membrane potential and greater DNA fragmentation, along with significantly lowering cell viability and proliferative capacity. Conclusions: These results confirm that UV radiation causes severe damages in skin cells, and they need to be protected from it, or it needs to be applied more cautiously, especially if the component used is UVB.

## 1. Introduction

The main factor for extrinsic skin ageing is UV exposure and, therefore, it is often called photoageing. The ageing of the skin by UV radiation is a cumulative process, and the rate of alteration depends on the wavelength, duration and intensity of solar exposure and the natural protection by skin pigmentation, as well as the activity of the immune system. The wavelengths of the solar UV radiation that reach the Earth’s surface are in a range from 290 to 400 nm. They can be divided into two bands, UVA (315–400 nm) and UVB (290–315 nm). The UVA/UVB ratio changes from cca 20/1 to more than 100/1 [1], depending on several factors, including the solar zenith angle, reflection by the soil, altitude and the presence of clouds or dust particles in the sky. UVB is more effectively absorbed by the atmosphere and, therefore, it reaches the maximum at overhead sun position [2,3]. Deeply penetrating UVA damages connective tissue in the dermis, while UVB penetrates only as far as the epidermis where it directly interacts with DNA, aromatic amino acids of proteins, NADH, NADPH, urocanic acid, eu- and pheomelanin, lipids and other endogenous chromophores [4]. Exposure to solar UV radiation can cause erythema, premature ageing of the skin, actinic keratosis, suppress the immune system and increase the risk of skin cancer [5]. Similar harmful effects can be observed with the use of UV artificial sources [6]. Currently used, commercially available UV tanning lamps emit primarily UVA with a minor part of UVB making up to approximately 4%. The equivalent UV irradiance of these appliances corresponds to midday tropical sun [7]. Other UV sources are used for therapeutic purposes, including psoralen-UVA photochemotherapy (PUVA) and UVB phototherapy. If we look at the total radiation power output of these devices, then the irradiance in W/m^2^ in the case of UVA sources is comparable to the maximum values measured from the sun but, in the case of UVB sources, this value is exceeded several times [8,9]. However, it is necessary to add that the photobiological effects of UV strongly depend on wavelength and, therefore, it is essential to work rather with the weighted (effective) quantities such as effective irradiance or effective dose, respecting the erythemal action spectrum of the Commission international de l’éclairage (CIE). No less important is also the fluence rate at which the total exposure dose is reached. Based on many studies, it has been found that short-term exposures to UV radiation from sunlamps, usually operating at 3–13 times higher fluence rates than summer sun, increases a risk of developing melanoma [10,11,12]. A variety of sources of UV radiation are used for medical purposes, emitting either broad-band UVA, broad- or narrow-band UVB or both components together [13]. In special cases, excimer monochromatic lasers can also be applied, especially for treatment of targeted skin areas [14,15,16]. Although the radiometric irradiances of all UV sources may be similar, they may differ significantly in the biological responses depending on their emission spectra, especially in cases where they contain a UVB component.

In the present study, we investigated phototoxic effects of two commercially available UV phototherapeutic sources on the human epidermal keratinocyte line HaCaT. We compared their effect on viability and five-day proliferative capacity, changes in ROS production, mitochondrial membrane potential and DNA damage.

## 2. Materials and Methods

### 2.1. Cell Culture and UV Irradiation 

The immortalized human keratinocytes HaCaT (CLS Cell Lines Service, Eppelheim, Germany) were grown in low glucose DMEM (Sigma-Aldrich, St. Louis, MO, USA) supplemented with sodium bicarbonate (3.7 g/L, Sigma-Aldrich, USA), fetal bovine serum (10% *v*/*v*, Biowest, Nuaillé, France), penicillin and streptomycin (100 U/mL and 100 μg/mL, resp., Sigma-Aldrich, USA) in darkness under a humidified atmosphere with 5% CO_2_ at 37 °C. For all experiments, HaCaT were trypsinized and transferred into 12-well plates at a total number of 1 × 10^5^ cells and cultivated for 24 h. The UV sources with relative homogeneous irradiance were made by placing 3 pairs of commercially available UV tubes in parallel so that the distances between the tubes were 3 mm. Actinic UVA PL-L 36W/10 (EAN: 8711500264817, Philips, The Netherlands, peak maximum at 370 nm and FWHM 25 nm, Figure S1) and narrow-band UVB PL-L 36W/01/4P (EAN: 8711500868893, Philips, The Netherlands, peak maximum at 310 nm and FWHM 6 nm, Figure S2) phototherapeutic tubes were used as the tubes for construction of the UVA and UVB sources, respectively. The irradiation of the cells took place at a distance of 20 cm from the tubes at an irradiance of 6.8 ± 0.4 mW/cm^2^ for UVA and 3.5 ± 0.4 mW/cm^2^ for the UVB source. We also measured emission spectra of those UV tubes and calculated their effective (CIE-weighted) irradiances using the erythemal weighting function [17]. The respective effective irradiances were 0.0062 mW/cm^2^ for the UVA source and 0.21 mW/cm^2^ for the UVB source. The effective dose (in J/m^2^) was then calculated as the time integral of effective irradiance. In skin photobiology or phototherapy, the commonly used unit for the effective dose is the minimal erythema dose (MED), where a value of 210 J/m^2^ was chosen as the 1 MED [18]. 

### 2.2. MTT Phototoxicity Test

Irradiation of the culture cells was performed in 0.6 mL/well phosphate-buffered saline (PBS) supplemented with 5 mM glucose (PBS-G). Irradiation was initiated with a 12-well plate, which was exposed to the highest dose (the longest exposure time). At certain time intervals, additional plates were added next to it so that, at the end of the irradiation procedure, the appropriate doses were reached for all plates. The maximum number of plates under one UV source was 3, which guaranteed sufficient homogeneity (see previous paragraph). Thus, apart from irradiation, all plates were exposed to similar conditions (withdrawal from the CO_2_ incubator, same incubation time in the irradiation medium). After irradiation, 4 parts of growth medium were added, and the cells were allowed to culture for the following days. From part of the samples (in particular, from 4 wells), the MTT test was performed on the first, second, third and fifth day. The MTT test was initiated by replacing the growth medium with fresh DMEM supplemented with 0.5 mg/mL methylthiazol tetrazolium bromide (MTT, Sigma-Aldrich) and, after another 4 h, the medium was completely removed and the wells with cell-produced formazan crystals were allowed to dry. On the fifth day, all formazan crystals were dissolved in 0.6 mL DMSO and absorbances were spectroscopically measured at 570 nm. The relative number of viable (oxidoreductase active) cells in the UV-treated and untreated samples was expressed as a percentage.

### 2.3. ROS Measurement

The increased production of the intracellular ROS upon exposure to UVA and UVB was analyzed using the fluorescence probe 5-(and-6)-chloromethyl-2′,7′-dichlorodihydrofluorescein diacetate (CM-H_2_DCFDA, Invitrogen by Thermo Fisher Scientific, Waltham, MA, USA). The cells were first incubated in PBS-G buffer supplemented with 10 μM CM-H_2_DCFDA for 20 min and then exposed to UVA or UVB radiation for 0, 2, 5, 10, 20, 40 and 80 min. After irradiation, fluorescence of CM-DCF was recorded by fluorescence reader Tecan Infinite 200pro (Tecan, Männedorf, Switzerland) at excitation and emission wavelengths 490 nm and 525 nm, respectively.

### 2.4. Mitochondrial Membrane Potential Measurement

Mitochondrial membrane potential was evaluated using the fluorescent probe 5,5′,6,6′-tetrachloro-1,1′,3,3′ tetraethylbenzimidazolylcarbocyanine chloride (JC-1, Biotium, Fremont, CA, USA). 

Formation of its J-aggregates is characterized by an increase of the red fluorescence, and it is favored at a higher mitochondrial membrane potential. After, UV irradiation in PBS-G, JC-1 was added to the cells at the final assay concentration of 2 μg/L, and the cells were allowed to incubate for 20 min. Then, the fluorescence emission intensity was measured using a fluorescence reader Tecan Infinite 200pro at excitation and emission wavelengths 490 nm and 590 nm, respectively.

### 2.5. Comet Assay

DNA damage caused by UVA or UVB radiation to the cells was investigated by a comet assay. Similar to previous measurements, the growth medium was replaced by PBS-G, and the cells were irradiated by means of the UVA or UVB sources for 2, 10 and 40 min. After irradiation, 4 parts of growth medium were added, and the cells were allowed to culture for 24 h. The comet assay was performed as previously described by Manisova and her co-workers [19]. The samples were finally stained by SYBR Green (Invitrogen, Thermo Fisher Scietific, Waltham, MA, USA) and manually scored using inverted fluorescence microscope Olympus IX 70 with CCD camera (Olympus, Tokyo, Japan) and CometScore 2.0 software (TriTek, Sumerduck, VA, USA). To make an evaluation of the DNA damage, we randomly chose approximately 200 cells from each given sample. Median values of the Olive tail moment (the fraction of tail DNA multiplied by the distance between the profile centers of gravity for DNA in the head and tail), the amount of the DNA in the head and in tail, were evaluated. 

### 2.6. Statistical Analysis

The data are shown as means ± standard errors for 8 (ROS measurement), 4 (MTT test, comet assay) or 3 (MMP measurement) independent experiments. One-way analysis of variance (ANOVA) was used for comparisons between experimental groups. In the case of the comet assay, we also evaluated normality of data distributions by the Shapiro–Wilk test and applied the Mann–Whitney U test using the software Statistica 13.4 (TIBCO Software Inc., Palo Alto, CA, USA). A *p* value of <0.05 was considered statistically significant for all tests.

## 3. Results

### 3.1. UVA and UVB Phototoxicity

**Figure 1:**
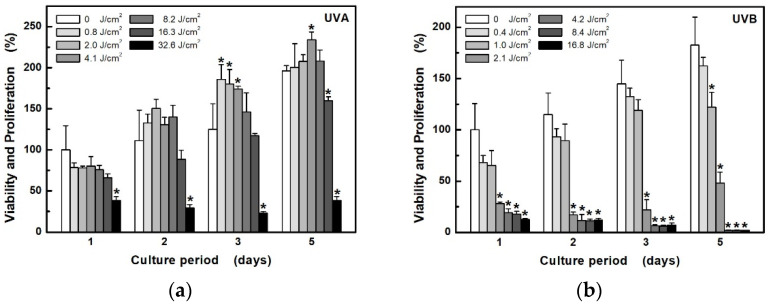
Development of HaCaT cell viability and proliferation capacity after exposure to different doses of UV radiation: (**a**) UVA at unweighted irradiances of 6.8 mW/cm^2^; (**b**) UVB at unweighted irradiances of 3.5 mW/cm^2^. Cell viability and proliferation were determined by measuring the activity of living cells using the MTT for the next 5 days. Values represent mean ± S.E. from 4 independent experiments. * Significant difference compared to non-irradiated cells (*p* < 0.05).

The MTT test was used in order to investigate the individual effect of both components of UV radiation on cell viability and the proliferative capacity of epidermal keratinocytes HaCaT. The effect depends not only on the type of radiation, but also on the dose (exposure time) (Figure 1). While longwave UVA shows changes in cell proliferation only at a high exposure dose (32.6 J/cm^2^, Figure 1a), UVB can already significantly damage cells at a 15 times lower dose (2.1 J/cm^2^, Figure 1b). In addition, it appears that low-dose UVA irradiation (up to about 4.1 J/cm^2^), after a statistically insignificant reduction in cell viability on the first day, can, in turn, stimulate their proliferation in the following days. 

### 3.2. ROS Production

**Figure 2:**
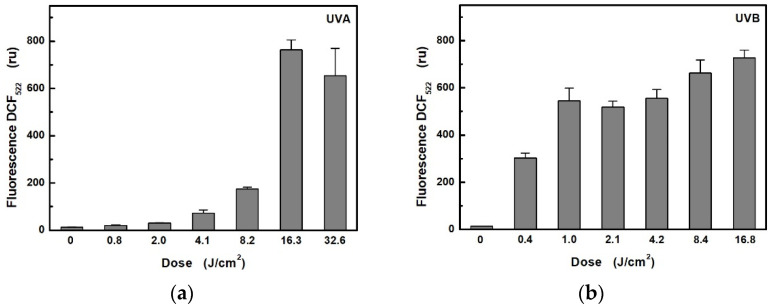
Dependence of ROS production in HaCaT cells on radiometric dose: (**a**) for UVA radiation at unweighted irradiances of 6.8 mW/cm^2^; (**b**) for UVB radiation at unweighted irradiances of 3.5 mW/cm^2^. Each value represents mean ± S.E. from 8 independent experiments.

The effect of ROS formation in HaCaT cells on different UVA and UVB exposure doses was investigated using a selective probe CM-H_2_DCFDA, which, on oxidation, becomes the highly green, fluorescent product (Figure 2). The obtained results showed significant differences in ROS production between UVA and UVB radiation. While UVB induced very fast ROS production, UVA irradiation, even at double irradiance (6.8 mW/cm^2^), led only to a slow increase in ROS. Twenty minutes of UVA irradiation (i.e., at a dose of 8.2 J/cm^2^) induced only approximately half the production of ROS as achieved using a UVB source at half the intensity within 2 min (i.e., at a dose of 0.4 J/cm^2^). A proportional increase in ROS production at higher doses for UVA (above 16.3 J/cm^2^) and UVB (above 1 J/cm^2^) was not observed, probably due to the saturation of the fluorescent probe, because, in control (non-irradiated) cells exposed to the additions of the oxidizing agent H_2_O_2_, a fluorescence intensity maximum of about 700 ru was reached (data not shown). 

### 3.3. Mitochondrial Membrane Potential

**Figure 3:**
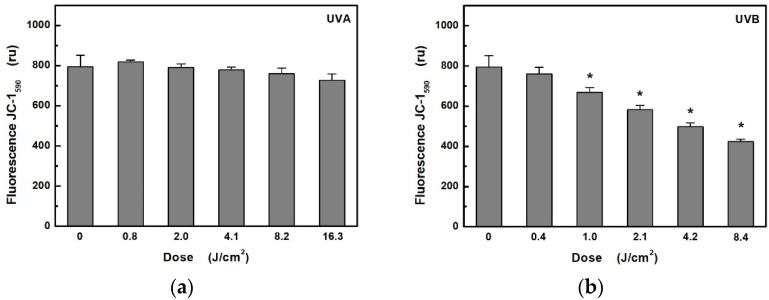
Effects: (**a**) of UVA; (**b**) UVB radiation on mitochondrial membrane potential in HaCaT cells were determined by measuring the fluorescence of JC-1 probe. Values represent mean ± S.E. from 3 independent experiments. Dose values correspond to unweighted exposure doses. * Significant difference compared to non-irradiated cells (*p* < 0.05).

The MMP assay using the JC-1 fluorescence probe was used to study changes of MMP in HaCaT cells exposed to UV radiation (Figure 3). In the case of UVB radiation, there was a significant decrease in MMP from exposure dose 1 J/cm^2^ (i.e., from 5 min at 3.5 mW/cm^2^). In contrast, UVA radiation did not cause any significant change even at 16 times the dose (i.e., 40 min at 6.8 mW/cm^2^). 

### 3.4. DNA Damage

**Figure 4:**
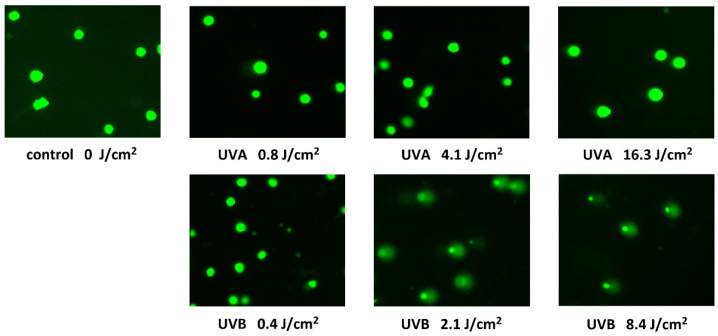
Fluorescence microscope images of the Comet assay acquired after 24-h exposure of HaCaT cells to UVA and UVB radiation for different unweighted doses. Images represent typical images of comets on dozens of microscope fields of view.

Single cell gel electrophoresis or comet assay is a sensitive and relatively simple method for detecting DNA fragmentation within cells [20]. The DNA levels in the head and tail, as well as the Olive tail moment, were calculated automatically using the CometScore 2.0 software. The DNA electrophoretic separation is not essentially normally distributed; therefore, in addition to the means and standard deviations, we determined the medians and the 25th and 75th percentiles. Due to the large data set, all evaluated parameters showed statistically significant differences from controls except for the Olive tail moments in the case of UVA exposure (Table 1). However, the comet assay revealed much greater increase in DNA fragmentation of HaCaT cells exposed to UVB radiation when, at the dose of 4.2 J/cm^2^, only a little more than 60% remained unseparated (Figure 4). 

## 4. Discussion

Approximately 5% of solar terrestrial radiation forms UV radiation. Total solar UV irradiance and the relative contributions of its components vary with the distance between the Earth and sun, the solar zenith angle, altitude, atmospheric gaseous and liquid constituents, as well as the presence of solid particles. If we study the amount of UV exposure, we must also take into account the reflection from the Earth’s surface, which, in the case of snow cover, can be up to 90% [21]. The solar zenith angle is actually the most important factor in determining the solar UV irradiance, especially UVB radiation, due to its attenuation by the ozone layer. As solar zenith angle increases, e.g., from 20° to 85°, the irradiance at 325 nm is decreased by a factor of 50, while that at 310 nm decreases by a factor of 500 and at 300 nm by a factor 3000 [22]. UV irradiance also depends on altitude. In general, it increases by about 4% per each 300 m [23]. During a sunny, summer day at the high solar zenith angle (around 70° and above), total solar UVA irradiance can reach values of about 3–6 mW/cm^2^ [22,24]. The UVB component maximally contributes only 1/20 up to 1/10 of the total solar UV irradiation [2,3]. However, if we take into account their photobiological effects, for example their erythemal action, then this ratio changes in favor of the UVB component. Considering the measurements performed by McKenzie et al. [24] and the erythemal weight function, then we can convert these values to the effective irradiances, which correspond to cca 6.7 μW/cm^2^ for UVA and 22 μW/cm^2^ for UVB at the solar zenith angle of 67.5°. 

In our work, we focused on the study of cell damage in human keratinocytes HaCaT exposed to UVA and UVB artificial sources, which are widely used in UV phototherapy. Whereas, in the case of the UVA source having the total irradiance of 6.8 mW/cm^2^, which corresponded with respect to emission and erythemal action spectrum to 6.2 μW/cm^2^ of effective irradiance, the artificial source for UVB showed relative higher irradiances and so exceeded several times the irradiance from the natural source. The total irradiance of the UVB source was 3.5 mW/cm^2^ and the appropriate effective irradiance 210 μW/cm^2^. However, it should be added, that in the case of UVB phototherapy, patients are exposed to radiation for a much shorter time, in the order of units of minutes and, therefore, the effective dose in a single therapeutic session does not have to exceed the daily solar UVB dose under suitable conditions [9,24]. The doses for UV phototherapy are commonly determined according to the Fitzpatrick skin type or MED [25]. In the case of UVB phototherapy based on MED, the initial dose is 0.5 MED, which then gradually increases during about 20 sessions to about 6 MED [26]. Radiation dosing for PUVA is similar. The treatment is initiated by the dose of 0.5–0.7 MED and then it is individually increased by a maximum of 60 percent per week [27]. The total number of sessions is usually, again, 20–25 [27]. The values for ambient UVB radiation in summer can be in the range of 10–25 MED per day for the latitudes of 50–20° N [28]. The minimal erythema dose of 10 MED is equivalent to 210 mJ/cm^2^ of the effective dose or exposure for 1000 s at the effective irradiance of 210 μW/cm^2^. In our in vitro assays, when HaCaT cells were exposed to the UVA source for 80 min (i.e., at the dose of 32.6 J/cm^2^ or recalculated effective erythemal dose of 29.8 mJ/cm^2^), there was a 62 percent decrease in their viability or proliferative capacity the following day. However, when using the UVB source at the half irradiance, a slightly greater effect (72 percent) was already observed after 10 min (i.e., at the dose of 2.1 J/cm^2^ or recalculated effective erythemal dose of 126 mJ/cm^2^). If we compare radiometric quantities, then UVB radiation is more harmful by at least 15 times (32.6/2.1). However, if we convert these values into effective doses, then UVB radiation appears to be less effective, which may indicate that the calculated effective doses correcting UV radiation according to the ability to induce skin erythema may not be fully related to phototoxic effects. Assuming that 5 min of cell irradiation by UVB did not produce statistically significant changes, then it appears that, in this case, calculations of effective doses can be overestimated 2–4 times in favor of UVB irradiation. Of course, another dose recalculation and evaluation could be performed if other action spectra were considered, e.g., for UV-induced carcinogenesis or DNA damage. Unfortunately, these are usually not completely delineated and, therefore, it is very difficult to apply them. However, it turns out that they can be very similar [29,30]. If we compare our results studying the effect of UV radiation on keratinocyte viability with other researchers, then in our case it was necessary to use much higher doses of UVB radiation to achieve similar effects. Dalmau et al. [31] found that a dose consisted of 728 mJ/cm^2^ UVA and 25 mJ/cm^2^ UVB was the maximum dose that did not cause any changes in HaCaT cell viability over the next 72 h. A similar value of non-phototoxic UVB dose for keratinocytes was reported in another recent publication [32]. However, it should be added that these studies used a broad-spectrum solar simulator Suntest CPS (Atlas Material Testing Technology, Chicago, IL, USA) or UVB Bio-Link Crosslinker BLX tubes (Vilber Lourmat, Collégien, France) emitting radiation in the range of 280–320 nm. In our case, a narrow-band phototherapeutic UVB PL-L 36W/01/4P tube (Philips, The Netherlands) with an emission maximum at 310 nm was used. The biological effect of UV radiation depends on the wavelength, which, in the case of the erythemal response to UVB radiation, can be very different. While at a wavelength of 310 nm, the value of the erythemal weighting factor is 0.074, at a wavelength of 300 nm it takes the value of 0.649 [18]. In addition, radiation in the region around 280 nm already interferes with the absorption spectrum of DNA molecules [33] and can thus significantly contribute to the reduction of cell viability even at lower doses. 

It is assumed that cell damage by UVA is primarily caused by the increase of ROS, which are recognized as mediators of DNA damage, mitochondrial dysfunction, alteration of intracellular communication, cellular senescence and degradation of the dermal extracellular matrix [34]. Our measurements showed that UVB radiation produces about 40 ((8.2 × 2)/(0.4)) times more ROS at the same radiometric doses. However, if we make the conversion to effective doses then, on the contrary, the yield of ROS production will be lower by about 0.6 ((6.2 × 1200 × 2)/(210 × 120)) times compared to the same effective dose of UVA radiation. 

In studying the effect of UV radiation on mitochondrial membrane potential or DNA of the HaCaT cells, we found that, in contrast to UVB, UVA did not cause depolarization or evident DNA fragmentation at a maximum radiometric dose of 16 J/cm^2^. UVB radiation is known to have a greater genotoxic effect than UVA radiation. In our measurement, there was a very significant (approximately 40 percent) fragmentation of DNA in HaCaT cells at the radiometric dose of 1 J/cm^2^ (i.e., at the effective dose of 63 mJ/cm^2^ or 3 MED). The same dose also led to a decrease in the mitochondrial membrane potential. Marabini et al. [35], using a broadband UVA lamp, observed significant DNA damage from a dose of 10 J/cm^2^. However, if a UVB lamp is used, then significant DNA damage is already measured at a dose of about 40 mJ/cm^2^ [35,36], i.e., at a 25 times smaller dose than in our case. However, it should be mentioned that a broad-band UVB source was used again and, in comparison with our source (peak at 310 nm, FWHM 6 nm), these sources (Triwood 31, Helios Italquartz, Italy or TL-20W/12 RS, Philips, The Netherlands) emit much more significantly shorter wavelengths (peak at 310 nm, FWHM 35 nm, or peak at 310 nm, FWHM 40 nm, respectively).

## 5. Conclusions

In summary, our results confirmed that UVB compared to UVA radiation is associated with significantly higher ROS production, decreased cell viability and proliferative capacity and impaired mitochondrial activity and damage to DNA. If we calculate radiometric doses into effective doses, then it may seem that UVB radiation has a much smaller impact on cell damage. However, it should be mentioned that the calculated effective doses reflect the response of the whole skin. The penetration of UV radiation in human skin is highly dependent on wavelength; while UVA reaches dermis, UVB is almost entirely absorbed by the epidermis [37]. As a consequence, this may lead to higher effective doses to induce changes associated with reduction in keratinocyte viability and proliferative capacity since a significant part of the UVB radiation is absorbed in the stratum corneum. In other words, if the upper layer of the skin was not present, which absorbs 90% of UVB, then the weighting factors for UVB would become an order of magnitude lower. Or, conversely, if significant loss of cell viability and DNA damage occurs at dose equivalents of 6 MED (10 min at effective irradiance of 0.21 mW/cm^2^) on the keratinocyte monolayer then, in the case of intact and whole skin, a similar effect can be achieved at doses 10 times higher. Furthermore, our results show that when studying the biological effects of UV radiation, the emission spectrum should be taken into account, especially if UVB radiation with the presence of shorter wavelengths (i.e., 280–300 nm) is applied. In addition, the wavelengths below 300 nm are already extremely well absorbed by the ozone layer [24] and do not participate in natural solar UV radiation at the Earth’s surface.

## Figures and Tables

**Table 1:** Statistical analysis of the comet assay for HaCaT cells exposed to UVA and UVB radiation for different unweighted doses. The evaluated parameters were DNA in head (%), DNA in tail (%) and Olive tail moment.

	Dose (J/cm^2^)	DNA in Head (%) Mean ± SD Median (25%; 75% Percentile)		DNA in Tail (%) Mean ± SD Median (25%; 75% Percentile)		Olive Tail Moment Mean ± SD Median (25%; 75% Percentile)	
control	0	93.78 ± 3.11		6.22 ± 3.11		0.97 ± 0.47	
		93.67 (91.67; 95.63)		6.33 (4.37; 8.32)		1.02 (0.71; 1.29)	
UVA	0.8	91.57 ± 5.82	*p* < 0.05	8.43 ± 5.82	*p* < 0.05	1.13 ± 1.30	*p* = 0.061
		92.34 (89.38; 94.71)	*p* < 0.05	7.66 (5.29; 10.61)	*p* < 0.05	1.02 (0.66; 1.31)	*p* = 0.760
	4.1	92.56 ± 6.28	*p* < 0.05	7.44 ± 6.28	*p* < 0.05	1.17 ± 1.47	*p* < 0.05
		93.05 (90.46; 96.03)	*p* < 0.05	6.95 (3.97; 9.54)	*p* < 0.05	1.07 (0.66; 1.41)	*p* = 0.171
	16.3	91.88 ± 13.63	*p* < 0.05	8.12 ± 13.63	*p* < 0.05	2.27 ± 5.42	*p* < 0.05
		95.61 (91.44; 99.97)	*p* < 0.05	4.39 (0.05; 8.62)	*p* < 0.05	0.91 (0.01; 1.49)	*p* = 0.113
UVB	0.4	89.37 ± 8.89	*p* < 0.05	10.63 ± 8.89	*p* < 0.05	1.84 ± 2.54	*p* < 0.05
		91.07 (87.23; 94.52)	*p* < 0.05	8.93 (5.48; 12.77)	*p* < 0.05	1.28 (0.89; 1.80)	*p* < 0.05
	2.1	61.31 ± 19.10	*p* < 0.05	38.69 ± 19.10	*p* < 0.05	10.70 ± 6.28	*p* < 0.05
		63.04 (47.37; 76.36)	*p* < 0.05	36.96 (23.64; 52.63)	*p* < 0.05	10.14 (6.21; 14.71)	*p* < 0.05
	8.4	*64.88 ± 14.64*	*p* < 0.05	*35.12 ± 14.64*	*p* < 0.05	8.68 ± 4.02	*p* < 0.05
		64.82 (54.67; 75.95)	*p* < 0.05	*35.18 (24.05; 45.33)*	*p* < 0.05	8.32 (5.71; 10.97)	*p* < 0.05

To compare the means or medians between the irradiated and non-irradiated (control) cells, one-way ANOVA or the Mann–Whitney test were applied, respectively. Values for which the distribution is Gaussian according to the Shapiro–Wilk test are written in italics.

## Data Availability

The data that support the findings of this study are available from the corresponding author upon reasonable request.

## Supplementary Materials

The following are available online at https://www.mdpi.com/article/10.3390/ijerph182312480/s1, Figure S1: Spectral radiation intensity distribution of the broad-band UVA source, Figure S2: Spectral radiation intensity distribution of the narrow-band UVB source.
