# Supplementary material for: Differences in the Effects of Broad-Band UVA and Narrow-Band UVB on Epidermal Keratinocytes
Source: Int J Environ Res Public Health. 2021 Nov 26;18(23):12480. doi: 10.3390/ijerph182312480 (PMC8656598; doi:10.3390/ijerph182312480)
Supplement: Supplementary file 1 [file ijerph-18-12480-s001.zip › ijerph-1460211-supplementary.pdf]

Differences in the Effects of Broad-Band UVA and Narrow-Band UVB on Epidermal Keratinocytes

Robert Bajgar [1,2,*], Anna Moukova [1], Nela Chalupnikova [1] and Hana Kolarova [1,2]

[1] Department of Medical Biophysics, Faculty of Medicine and Dentistry, Palacky University in Olomouc, Hnevotinska 3, 775 15 Olomouc, Czech Republic; anna.moukova01@upol.cz (A.M.); nela.chalupnikova01@upol.cz (N.C.); hana.kolarova@upol.cz (H.K.)
[2] Institute of Molecular and Translational Medicine, Faculty of Medicine and Dentistry, Palacky University in Olomouc, Hnevotinska 3, 775 15 Olomouc, Czech Republic
* Correspondence: robert.bajgar@upol.cz; Tel.: +420-585-632106

Supplementary Materials:
Spectral radiation distribution of the used UV sources

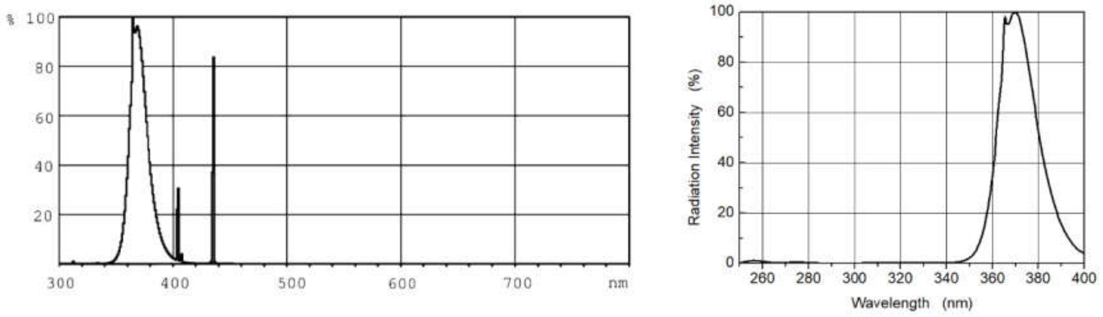

(**a**) (**b**)

Figure S1. Spectral radiation intensity distribution of the broad-band UVA source: (**a**) declared by the manufacturer (https://www.assets.signify.com/is/content/PhilipsLighting/fp927903421007-pss-global); (**b**) measured by the spectrofluorometer FLS980 (Edinburgh Instruments, UK)

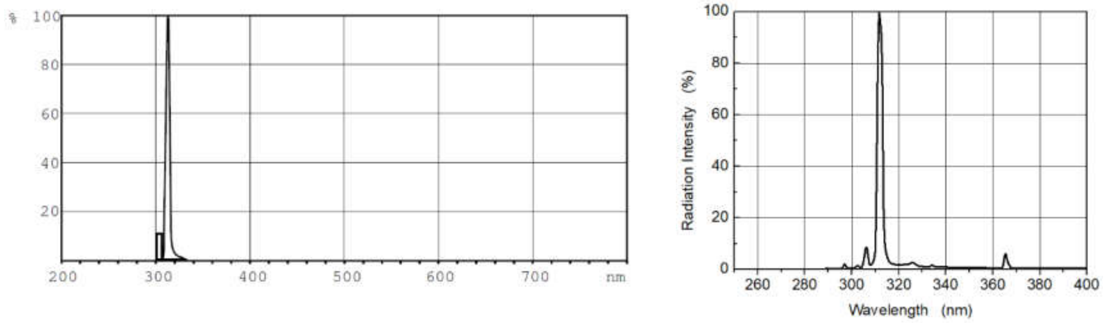

(**a**) (**b**)

Figure S2. Spectral radiation intensity distribution of the narrow-band UVB source: (**a**) declared by the manufacturer (https://www.assets.signify.com/is/content/PhilipsLighting/fp927903400121-pss-global); (**b**) measured by the spectrofluorometer FLS980 (Edinburgh Instruments, UK)
